# Deep Learning and Multidisciplinary Imaging in Pediatric Surgical Oncology: A Scoping Review

**DOI:** 10.1002/cam4.70574

**Published:** 2025-01-15

**Authors:** M. A. D. Buser, J. K. van der Rest, M. H. W. A. Wijnen, R. R. de Krijger, A. F. W. van der Steeg, M. M. van den Heuvel‐Eibrink, M. Reismann, S. Veldhoen, L. Pio, M. Markel

**Affiliations:** ^1^ Princess Máxima Center for Pediatric Oncology Utrecht The Netherlands; ^2^ Wilhelmina Children's Hospital University Medical Center Utrecht Utrecht The Netherlands; ^3^ Department of Pediatric Surgery Charité‐Universitätsmedizin Berlin Berlin Germany; ^4^ Department of Pediatric Radiology Charité‐Universitätsmedizin Berlin Berlin Germany; ^5^ Pediatric Surgery Unit Université Paris‐Saclay, Assistance Publique‐Hôpitaux de Paris, Bicêtre Hospital Le Kremlin‐Bicêtre France

## Abstract

**Background:**

Medical images play an important role in diagnosis and treatment of pediatric solid tumors. The field of radiology, pathology, and other image‐based diagnostics are getting increasingly important and advanced. This indicates a need for advanced image processing technology such as Deep Learning (DL).

**Aim:**

Our review focused on the use of DL in multidisciplinary imaging in pediatric surgical oncology.

**Methods:**

A search was conducted within three databases (Pubmed, Embase, and Scopus), and 2056 articles were identified. Three separate screenings were performed for each identified subfield.

**Results:**

In total, we identified 36 articles, divided between radiology (*n* = 22), pathology (*n* = 9), and other image‐based diagnostics (*n* = 5). Four types of tasks were identified in our review: classification, prediction, segmentation, and synthesis. General statements about the studies'’ performance could not be made due to the inhomogeneity of the included studies. To implement DL in pediatric clinical practice, both technical validation and clinical validation are of uttermost importance.

**Conclusion:**

In conclusion, our review provided an overview of all DL research in the field of pediatric surgical oncology. The more advanced status of DL in adults should be used as guide to move the field of DL in pediatric oncology further, to keep improving the outcomes of children with cancer.

## 1 Introduction

In Europe, more than 14,000 children are diagnosed with cancer every year [1]. Besides hemato‐ and neuro‐oncologic entities, extracranial solid tumors account for approximately 38% of the cases, which represent a field of responsibility for medical professionals working in pediatric surgical oncology [1, 2]. To diagnose and treat solid tumors, medical images play an important role, including radiology, pathology, and other image‐based diagnostics.

Radiology is an important cornerstone in the management of children with cancer, from diagnosis and response assessment, as well as surveillance for relapses in the follow‐up [3]. Imaging modalities such as X‐ray and ultrasound (US) are fast and often used for the initial diagnosis of pediatric tumors [4]. Computed tomography (CT) and magnetic resonance imaging (MRI) provide detailed anatomical information, useful for discrimination of tumor types, staging, volume assessment, and organ infiltration and intravascular extension, as well as response monitoring and surgical and radiotherapy planning [5]. Furthermore, nuclear imaging such as positron emission tomography/CT (PET‐CT) and meta‐iodobenzylguanidine (MIBG) imaging can aid in the diagnosis, staging, and monitoring of specific tumors [6, 7].

Pathology is still the gold standard in many tumor types for diagnostician and risk stratification [8, 9, 10]. Since the introduction of whole slide imaging, the amount of visual pathological data has widely increased [11]. Combined with the rise of precision medicine and the associated need for more precise tumor subtyping, an increased burden is placed upon pathologists to process visual information in a timely and thorough manner while limiting the large inter‐ and intra‐observer variability.

Medical visual information is not limited to the often recognized areas of radiology and pathology. In clinical practice, image‐based diagnostics also play an important role. From skin pictures in dermatological oncology and 3D images of children to screen for genetic predispositions, to fundoscopic images, more and more visual data are being created and used [12, 13, 14].

Although technical advances in radiology, pathology, and other image‐based diagnostics have improved diagnosis and outcomes for children with cancer, early detection and treatment of certain aggressive malignancies remain challenging. The increased amount of medical images in different disciplines combined with their specific challenges indicates a need for implementation of advanced image processing technology, such as artificial intelligence (AI). This might not only decrease workload by automation but also give insights beyond human comprehension.

**FIGURE 1:**
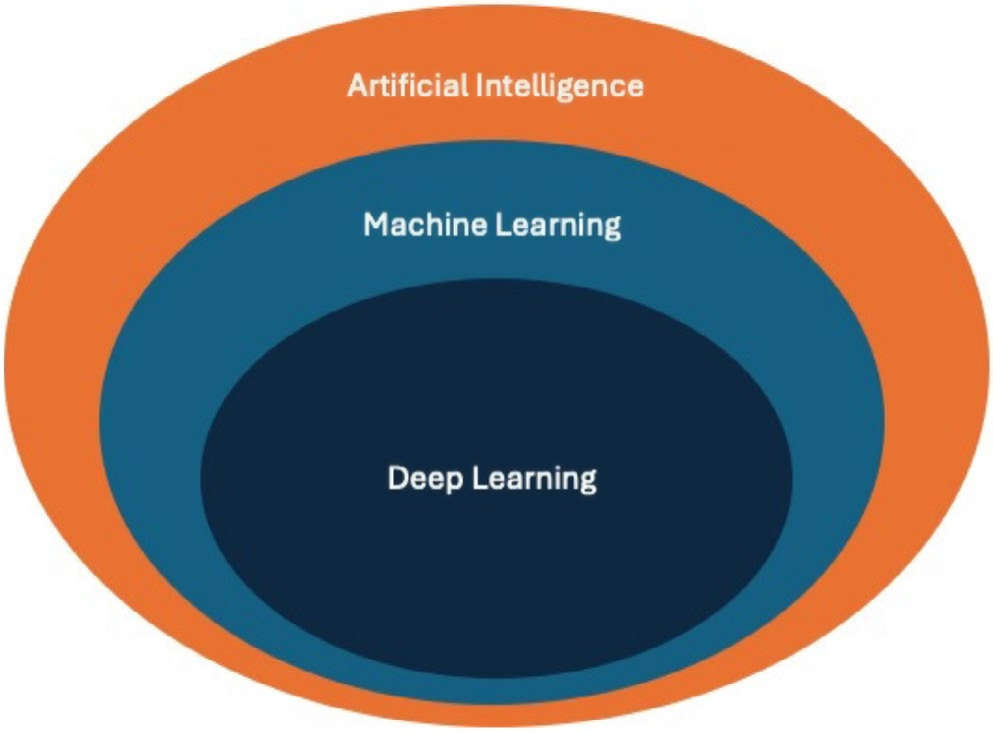
Overview of artificial intelligence and its relation to deep learning.

AI applications are gaining more interest in the medical community [15]. In machine learning (ML), a subfield of AI, tasks are automated by utilizing computational models that are able to find patterns using training data, and generalize these patterns to unseen data. An emerging field in medical image analysis is DL, a subfield of ML where models consist of many computational layers (Figure 1). These layers can transform the input data to the relevant output parameter(s). As DL is a powerful tool, this is especially helpful in the information‐rich context of images. In adult oncology, DL is already widely applied both in research and in the clinical workflow [15, 16, 17, 18, 19, 20].

In our scoping review, we aim to introduce the reader to the current status of DL applications specifically for use in medical images in pediatric surgical oncology. First, we will give an introduction about the terms most commonly used in DL. Then, we will discuss the spectrum of DL applications we found and highlight the most important areas of current research and those where opportunities can still be seized.

## 2 Background

We will briefly introduce the terms used, both to familiarize the reader with these terms and to give strict definitions to increase consistency in our review. Deep learning is based on a group of algorithms called neural networks (NNs) [21]. A subtype of NNs especially suited for image‐related tasks are convolutional neural networks (CNNs), loosely inspired by the visual cortex [22]. The exact inner workings of (C)NNs fall beyond the scope of this review.

DL methods need to be “trained” in order to be able to perform the specific task. This training is usually based on a dataset including pairs of the input together with the desired and already known output (often referred to as “ground truth”). Besides a training dataset, common DL methods use a testing dataset (consisting of unseen examples), to ensure a fair evaluation of the DL method. The testing dataset can either be from the same source as the training dataset (commonly referred to as test set) or from another data source (an external test set).

Different image‐related tasks can be performed by DL, with different types of outcome parameters. We will define these tasks as technical tasks in our review, to distinguish them from the clinical embedding. We will discuss the technical tasks included in our review, but more tasks may exist.

### 2.1 Classification and Prediction

Classification can be regarded as the most basic of DL tasks [23]. The most elemental form, binary classification, divides the input examples into two groups. Classification can be applied to a wide range of input types, for example numerical data such as patient parameters, 2D images such as pathology images or 3D volumes such as CT images. Often, binary classification distinguishes between positive and negative cases, for example whether disease is present or not. Classification can also be multiclass, such as trying to predict the specific subtype of a disease.

In the literature, classification is often referred to as prediction. However, the output of classification is discrete (e.g., tumor subgroups), whereas the output of prediction is continuous (e.g., event free survival). We will use this distinction in our review, even if not consistent with the terminology of the specific study.

The most important and easiest to interpret outcome parameters in classification are accuracy, sensitivity and specificity [24]. Accuracy is defined as the number of correct predictions divided by the total number of predictions, implying that an accuracy of 100% is the perfect score. Sensitivity is defined as the number of true positives divided by the total number of positive examples. Specificity is defined as the number of true negative cases divided by the total number of negative examples. These scores can be combined into a metric called the Area Under the Receiver Operating Characteristic Curve (AUC‐ROC). This metric represents the trade‐off between sensitivity and specificity, providing an overall measure of the model's performance. An AUC‐ROC score of 1 indicates perfect performance.

### 2.2 Semantic Segmentation

Semantic segmentation is an important task when using DL in images [25]. In essence, segmentation is pixel‐wise classification of an image. By classification of every pixel, an image is divided into discrete parts. Again, this can be binary, such as segmenting an X‐ray image into tumorous/nontumorous areas, or multiclass, for example the segmentation of organs at risk on a CT scan in radiotherapy planning.

Segmentation algorithms are almost always validated by comparing the resulting segmentation to the manually created ground truth. The most important outcome score in segmentation applications is the Dice similarity coefficient (Dice) [26]. A Dice score of 0 can be interpreted as no overlap between the segmentation and the ground truth, while a Dice score of 1 means perfect overlap. Other validation parameters of segmentation do exist and can provide additional information about the quality of the segmentation, but for our review, we will only focus on the most common and most intuitive Dice score.

### 2.3 Image Synthesis

Image synthesis is the process of creating an artificial image based on a provided input [27]. The input can be text, for example creating an image based on a word of sentence, or another image, such as creating a T1‐weighted MR image from a T2‐weighted MR image.

There are several ways to evaluate image synthesis [28]. The most used outcome score is the mean absolute error between the ground truth image and the predicted image. A higher error indicates a worse outcome. If CT scans are used as the ground truth image and the predicted image, the error is often expressed in Hounsfield Units (HU), a standardized unit to express radiodensity in CT images.

### 2.4 AI in the Clinical Workflow

Roughly, adding DL to the multidisciplinary workflow of oncological care can be divided in two use cases. First, the DL can be used to assist a medical specialist in their work. For example, performing tumor volume measurements on MRI can be done by the radiologist, but using DL can make this faster and more reliable. Second, DL can be used to augment the specialist, by adding another layer of information. For this, you can think about defining DL‐based risk scores. In case of augmenting, the oncologist plays an important role in interpreting this extra information of the context of the whole patient and treatment plan. The exact implementation in the clinical workflow therefore depends on the exact use case of the application.

## 3 Methods

This systematic review consists of three sections, all focusing on a different part of the question “What is the role of deep learning for medical image analysis in the diagnosis and treatment of pediatric surgical oncology?” To structure our review, we defined three areas of interest, based on the specific image type: radiology including radiotherapy, pathology, and other image‐based diagnostics. All sections are based on the same search, but with different inclusion and exclusion criteria.

### 3.1 Search

The systematic search of the literature was conducted in October 2023, using PubMed, Scopus, and Embase. The search string was developed and adapted for each specific database in collaboration with a librarian. The resulting strings can be seen in Table S1. Each database was searched based on title, abstract, and keywords. The search results from the databases were merged and duplicates were removed.

### 3.2 Inclusion and Exclusion Criteria

Studies about the application of DL in pediatric, extracranial solid tumors were included in the review. The full list of the in‐ and exclusion criteria can be seen in Table 1.

**TABLE 1:** List of in‐ and exclusion criteria for the review in general and per subsection.

Criteria type	Section	Description
Inclusion	General	Studies about extracranial, pediatric solid tumors
Inclusion	General	Studies about DL
Exclusion	General	Median age not reported in a non‐childhood specific tumor
Exclusion	General	Median age > 18 in a non‐childhood specific tumor
Exclusion	General	Classical ML
Exclusion	General	No peer‐review or original study
Inclusion	Section 4.1	Study about radiology
Inclusion	Section 4.2	Study about pathology
Inclusion	Section 4.3	Study met general inclusion criteria, but not inclusion criteria for Sections 4.1 or 4.2

### 3.3 Screening

Screening was performed for each section separately. Preliminary screening was done in ASReview (Version 1.2.1.), based on title and abstract [29]. ASReview uses a process called “active learning” to rank studies on relevance during the review process, updating the order of the studies to be screened with every study that is in‐ or excluded. To initialize the screening order, the screener first provides three relevant and three irrelevant articles, based on their experience in the topic. Because the screener reads more relevant studies first, screening can be stopped when meeting predefined stopping criteria. We decided to combine two common, literature‐based, stopping criteria [30]. First, a screening percentage of 33% of the total number of studies had to be reached. After this was fulfilled, screening continued until 50 studies were consecutively labeled irrelevant. Screening was done in duplicate by a technical physician (M.A.D.B.) and a pediatric surgeon (M.M.). As a result of the changing screening order while using ASReview, there is a potential difference in the studies that are seen by each screener. Therefore, nonconsensus was defined as either one screener did include the study and the other screener did not, or the study only being included by one screener and not seen by the other. After the first screening, consensus was reached by discussing the nonconsensus studies. If one screener excluded the study and the other screener did not label it, we implicitly assume consensus for noninclusion.

The studies selected after the title and abstract screening were reviewed in full text for the final inclusion. Again, consensus was reached by discussing the studies in case of nonconsensus. When nonconsensus could not be resolved, one of the coauthors was asked to provide a third opinion. The citations of all included studies were checked to include relevant papers not present in the original search.

### 3.4 Data Extraction

The following data were extracted from the included studies: technical task, clinical aim of the study, tumor type, anatomical region, study cohort, number of patients and number of samples, the DL model used, the type of validation (in case of an external dataset, only the external dataset was mentioned), the validation metrics and the validation performance. In case of multiple validated DL methods, the highest scoring method was reported.

For Section 4.1, imaging modality was also extracted. For Section 4.2, the staining used and highest magnification was included. For Section 4.3, the image type was reported.

## 4 General Results

**FIGURE 2:**
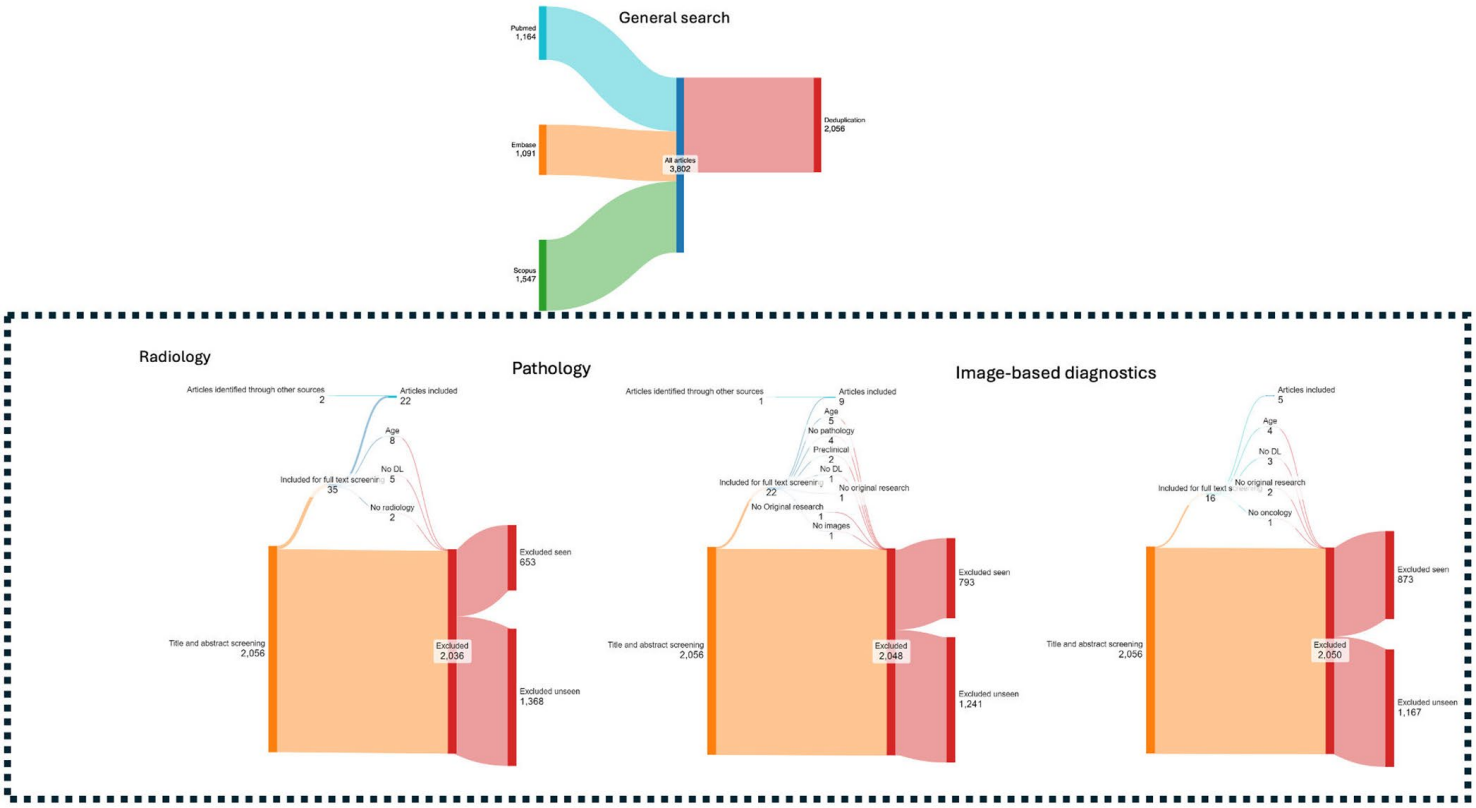
Flow diagram of inclusions for all three subquestions.

In total, 3802 studies were identified (1164 from Pubmed, 1091 from Embase and 1547 from Scopus). After deduplication, 2056 studies remained (Figure 2).

### 4.1 Deep Learning in Pediatric Oncology Radiology and Radiotherapy

In total, 22 studies about DL in (nuclear) imaging were selected. Most studies focused on segmentation (*n* = 10) or classification (*n* = 8). Other research areas included image synthesis (*n* = 3) or prediction (*n* = 1). Most studies used (CB)CT (*n* = 8) or MRI (*n* = 9) as input modality. Other modalities included X‐ray (*n* = 3), US (*n* = 1), or MIBG imaging (*n* = 1).

#### 4.1.1 Classification and Prediction

Eight studies in our review focused on classification and one on prediction. Most studies focused on classifying imaging between benign and malignant. Breden et al. showed a sensitivity of 89.1% and a specificity of 93.2% to classify X‐rays of pediatric knees suspected to be benign or malignant [31]. They used a dataset including the X‐ray images of 176 children (*n* = 366 X‐ray images) with a histopathologically confirmed bone tumor and 220 X‐ray images of healthy children. To further support their goal of aiding less experienced physicians in their decision making, they implemented Explainable AI (XAI) to give insight into what part of the image was used to base this classification on.

Hinterwimmer et al. [32] also focused on bone tumors, as they used transfer learning to classify X‐rays of extremities between Ewing sarcoma and acute osteomyelitis. Transfer learning is a way of leveraging training on a large, often unrelated, dataset (pre‐training) to use for a task in which limited data are available (fine‐tune training). Often, the technical task for pretraining and fine‐tune training is the same. Hinterwimmer used > 42,000 unlabeled X‐ray images of the musculoskeletal region for pretraining, in which the task was to cluster these unlabeled X‐ray images in five groups that were most similar. Next, they performed fine‐tuning training on 63 images of 22 acute osteomyelitis and 41 osteosarcoma patients, using histopathologically confirmed ground truth. This two‐staged strategy resulted in a classification accuracy of 88.1%.

Yang et al. [33] investigated thyroid nodules on US, for which the malignancy was histologically confirmed with a biopsy or surgery in a group of 139 patients. They used a previously published DL method, developed on an adult dataset, with a resulting accuracy of 87.5% for the classification between benign and malignant nodules [34]. They compared this with three radiologists who also classified the US images with a mean accuracy of 58.3% (31.2%–75.0%). Although the accuracy and specificity of the DL algorithm was comparable in both the adult series and the pediatric series, the pediatric population showed a decreased specificity. Therefore, the authors did not recommend the use of the algorithm into pediatric clinical practice yet.

Other studies focused on classification of specific diagnoses. Zhu et al. [35] trained a DL method on abdominal CT scans of 364 children with a renal tumor, to discriminate between Wilms tumor and non‐Wilms tumor. The ground truth was histopathologically determined. The test set was also classified by three radiologists with 3–15 years of experience, resulting in a sensitivity ranging from 13.3% to 20.0% between the radiologists. The resulting DL method scored a significantly higher sensitivity of 78.1%.

Banerjee [36] used a combination of DWI and T1‐weighted MRI scans to classify rhabdomyosarcoma patients in suspected embryonal and suspected alveolar subtype (confirmed by biopsy). To mitigate the effect of their limited number of patients (*n* = 21), they used transfer learning with a big dataset of nonmedical images. Their method was evaluated by cross‐validation, resulting in a classification accuracy of 85%.

Yang, Zhou, and Li [37] developed a classification method based on MRI to discriminate children with either hemangioma or hepatoblastoma. They compared this method with a classification method based on the clinical parameters age, gender, hepatic function, and serum alpha fetoprotein levels. The DL method based on the MR imaging performed better with an accuracy of 77%, compared with 60% for the clinical prediction model. However, the statistical significance of this difference was not reported.

Consalvo et al. [38] tried to classify between acute osteomyelitis and osteosarcoma. They did a two‐phased approach in which they first trained a DL algorithm to classify images in pathological and nonpathological X‐rays. Next, they used another DL network to classify the pathological images between acute osteomyelitis and osteosarcoma, with a final accuracy of 86.7%.

Mayampurath [39] used MIBG scans obtained before chemotherapy to classify patients between likely and not likely to react to induction chemotherapy (Curie score ≤ 2). When comparing a clinical model with the DL method, they found that the classification methods did not significantly differ in performance (56% for the DL method, 66% for the clinical method).

Guerreiro [40] focused on prediction instead of classification, namely predicting dose distributions for both proton and photon radiation in pediatric abdominal tumors. An extensive model, using 10 different inputs including the planning CT and segmentations of several structures, was used to predict the patient‐specific radiotherapy dose. Besides a technical evaluation, they performed a study in which radiologists needed to state their preference for either proton or photon radiation, based on either the planned or predicted dose estimation. For 18 of the 20 patients in this evaluation, the choice was the same for the predicted and planned dose.

#### 4.1.2 Semantic Segmentation

Out of the 10 included studies, most studies focused on tumor segmentation only. Kayal et al. [41] compared several methods of segmentation of osteosarcoma on diffusion‐weighted imaging (DWI). Their included methods contained both classical ML techniques as well as one DL method. Their DL method, trained on a total of 55 patients, showed comparable results to the other methods with a mean Dice score of 0.73 (Table 2).

**TABLE 2:** Table of inclusions for radiological studies.

Title	First author	Year	Task	Clinical focus	Modality	Tumor type
Deep Learning‐Based Detection of Bone Tumors around the Knee in X‐rays of Children	Breden	2023	Clas	Malignant/non‐malignant	X‐ray	Bone tumors
From Self‐Supervised Learning to Transfer Learning with Musculoskeletal Radiographs	Hinterwimmer	2022	Clas	Ewing sarcoma/acute osteomyelitis	X‐ray	Ewing sarcoma
MRI‐Based Deep Learning Model for Differentiation of Hepatic Hemangioma and Hepatoblastoma in Early Infancy	Yang	2023	Clas	Hepatoblastoma/hepatic hemangioma	MRI	Liver tumors
CT‐Based Identification of Pediatric Non‐Wilms Tumors Using Convolutional Neural Networks at a Single Center	Zhu	2023	Clas	Wilms/non Wilms tumor	CT	Renal tumors
Transfer Learning on Fused Multiparametric MR Images for Classifying Histopathological Subtypes of Rhabdomyosarcoma	Banerjee	2017	Clas	Histopathological subtypes of rhabdomyosarcoma	MRI	Rhabdomyosarcoma
Thyroid Nodules on Ultrasound in Children and Young Adults: Comparison of Diagnostic Performance of Radiologists' Impressions, ACR TI‐RADS, and a Deep Learning Algorithm	Yang	2022	Clas	Malignant/non malignant	US	Thyroid
Two‐Phase Deep Learning Algorithm for Detection and Differentiation of Ewing Sarcoma and Acute Osteomyelitis in Paediatric Radiographs	Consalvo	2022	Clas	Ewing sarcoma/acute osteomyelitis	X‐ray	Intraosseous Ewing sarcoma
Predicting Response to Chemotherapy in Patients With Newly Diagnosed High‐Risk Neuroblastoma	Mayampurath	2021	Clas	Prospective chemotherapy response	MIBG	Neuroblastoma
Deep Learning Prediction of Proton and Photon Dose Distributions for Paediatric Abdominal Tumours	Guerreiro	2020	Pred	Photon and proton dose distribution prediction	CT	Wilms tumor, Neuroblastoma
Segmentation of Osteosarcoma Tumor Using Diffusion Weighted MRI: A Comparative Study Using Nine Segmentation Algorithms	Kayal	2019	Seg	Tumor/background	MRI	Osteosarcoma
Comparative Multicentric Evaluation of Inter‐Observer Variability in Manual and Automatic Segmentation of Neuroblastic Tumors in Magnetic Resonance Images	Veiga‐Canuto	2022	Seg	Tumor/background	MRI	Neuroblastoma
Independent Validation of a Deep Learning nnU‐Net Tool for Neuroblastoma Detection and Segmentation in MR Images	Veiga‐Canuto	2023	Seg	Tumor/background	MRI	Neuroblastoma
Benchmarking Wilms' Tumor in Multisequence MRI Data: Why Does Current Clinical Practice Fail? Which Popular Segmentation Algorithms Perform Well?	Müller	2019	Seg	Tumor/background	MRI	Wilms tumor
Radiologic Versus Segmentation Measurements to Quantify Wilms Tumor Volume on MRI in Pediatric Patients	Buser	2023	Seg	Tumor/background	MRI	Wilms tumor
Segmentation of Deformed Kidneys and Nephroblastoma Using Case‐Based Reasoning and Convolutional Neural Network	Marie	2019	Seg	Tumor/background	CT	Wilms tumor
Fusion of Multiple Segmentations of Medical Images Using OV(2)ASSION and Deep Learning Methods: Application to CT‐Scans for Tumoral Kidney	Corbat	2020	Seg	Tumor/kidney	CT	Wilms tumor
Multi‐View Convolutional Neural Networks for Automated Ocular Structure and Tumor Segmentation in Retinoblastoma	Strijbis	2021	Seg	Tumor/anatomical eye structures	MRI	Retinoblastoma
Auto‐Segmentation of Important Centers of Growth in the Pediatric Skeleton to Consider During Radiation Therapy Based on Deep Learning	Qiu	2021	Seg	Skeletal growth centers/background	CT	Not tumor specific
Prediction of MYCN Gene Amplification in Pediatric Neuroblastomas	Yeow	2023	Seg	Tumor/background	CT	Neuroblastoma
Deep Learning‐Enabled MRI‐Only Photon and Proton Therapy Treatment Planning for Paediatric Abdominal Tumours	Florkow	2020	Synth	MRI to CT	MRI	Wilms tumor, Neuroblastoma
Deep Learning Based Synthetic CT From Cone Beam CT Generation for Abdominal Paediatric Radiotherapy	Szmul	2023	Synth	CBCT to CT	CBCT	Not tumor specific
Training a Deep Neural Network Coping With Diversities in Abdominal and Pelvic Images of Children and Young Adults for CBCT‐Based Adaptive Proton Therapy	Uh	2020	Synth	CBCT to CT	CBCT	Not tumor specific

Abbreviations: CBCT, cone beam computed tomography; Clas, classification; CT, computed tomography; MRI, magnetic resonance imaging; Pred, prediction; Seg, segmentation; Synth, synthesis. The greyish color supports the visual differentation of the different tasks (Classification, Prediction, etc..).

A multicenter study about segmentation of neuroblastoma was performed by Veiga‐Canuto et al. [42]. They included 132 patients from five centers in Europe and compared the results of the segmentation algorithm with the interobserver variability. They showed Dice scores above 0.96. In the next study by Veiga‐Canuto, the neuroblastoma segmentation method was tested on an independent test set of 300 children [43]. For this test set, they found a median Dice score of 0.98.

Three studies focused on Wilms tumor segmentation. First, Müller et al. [44] aimed to set a benchmark dataset for Wilms tumor segmentation, consisting of 28 MRI scans from 17 patients. They compared several classical ML algorithms with one DL method. In this case, several of the classical ML methods outperformed the DL (Dice = 0.30 postchemotherapy scans), which was attributed to the small dataset they used to train the network. Buser et al. [45] used a larger set of patients and a more advanced DL method, leading to a postchemo Dice score of 0.90. The aim of their study was to determine whether DL can support the radiologist in making more accurate tumor volume measurements.

Marie et al. [46] proposed a semiautomatic workflow to segment healthy kidney and tumor in Wilms tumor patients. In their method, the DL method was not trained on intact MRI scans of multiple patients, but on individual patient slices for each patient separately. Several slices belonging to one patient were manually segmented, after which a DL method is trained specifically for this patient. They showed that with manual segmentation 26% of the tumor slices, a mean segmentation Dice of 0.897 can be reached. However, this system was not fully automated and did need input from a radiologist. Corbat [47] used the same DL method for Wilms tumor patients, but for both kidney and tumor segmentation. They developed a fusion DL method to determine if a pixel was tumor or kidney if the DL segmented that pixel as both tumor and kidney. They showed an increased segmentation performance when using this fusion method, with a Dice score increasing from 0.86 to 0.88 for kidney and tumor combined.

Yeow et al. [48] used classical ML methods to try and predict MYCN gene amplification neuroblastoma cases from abdominal CT scans. However, part of their pipeline was a DL‐based segmentation method. For training, they used a set of 47 patients, which resulted in neuroblastoma segmentation with a median Dice score of 0.68. The rest of the pipeline, with the goal of predicting MYCN gene amplification, included extraction of image features of the segmented tumor and several ML classifiers, with a highest overall accuracy of 87.88%.

Strijbis et al. [49] not only focused on the tumor segmentation on the MR scans of retinoblastoma patients but also included segmentation of relevant anatomical structures located in the eye. The tumor segmentation was segmented with a median Dice score of 0.78. Next to tumor segmentation, they segmented four other structures, with Dice scores ranging from 0.64 for retinal detachment to 0.87 for the sclera.

Qiu et al. [50] did not focus on tumor segmentation. Instead, they developed a DL method to segment growth centers in the skeleton, with the aim of sparing these centers during radiotherapy. Besides a technical validation, they compared radiotherapy plans with and without avoidance of skeletal growth centers, showing the clinical need for their research.

#### 4.1.3 Image Synthesis

All image synthesis studies (*n* = 4) we identified were within the field of radiotherapy. These studies all aimed to create synthetic CTs to perform radiotherapy planning. Florkow [51] included images of 66 children with either Wilms tumor or neuroblastoma to train a DL method to create synthetic CT scans from MR images. Besides technical evaluations, they quantified the effect of using a synthetic CT instead of a ground truth CT on the resulting dose distribution of the radiotherapy plan. They defined clinically acceptable differences between the dose distribution of the ground truth CT and the synthetic CT to be 2% or less, which were obtained in 61/66 patients.

Cone beam CT (CBCT) was used as input in the studies of Szmul and Uh. Szmul et al. [52] trained their method on 63 patients with abdominal, thoracic, and pelvic region of radiation. They reported a mean absolute error between the ground truth CT and the synthetic CT of 55 HU. Uh et al. [53] also used CBCT to generate synthetic CT scans. They excluded air in the gastrointestinal tract from their analysis, leading to slightly smaller mean absolute errors of 47 HU. They also performed a patient‐by‐patient dosimetric evaluation of the synthetic CT compared with the reference CT.

### 4.2 Deep Learning in Pediatric Oncological Pathology

In total, nine studies focusing on DL in pathology were included. Most studies were focused on classification (*n* = 7). One study was focused on prediction, and one study was focused on segmentation.

#### 4.2.1 Classification and Prediction

Three studies focused on (ganglio‐)neuroblastoma. Liu et al. [54] aimed to classify neuroblastoma patients with the intent to discriminate between favorable and unfavorable histology. They compared several methods, of which the DL method showed an accuracy of 90.9%. The study by Gheisari et al. [55] focused on classification between differentiated and undifferentiated neuroblastoma, ganglioneuroblastoma, and ganglioneuroma. They compared their methods with several other, commonly used, DL networks, for which they found a significantly different precision of 84%. Liu, Yin, and Sun [56] also created a method to classify whole slide images to discriminate between neuroblastoma, ganglioneuroblastoma, and ganglioneuroma. Besides a technical validation, they performed an experiment to determine whether diagnostic accuracy of seven predefined classes improves when a junior pathologist worked in conjunction with the developed DL tool. They found a diagnostic accuracy of 56% without the DL tool, and 75.86% with the DL tool.

Frankel et al. [57] focused on the differential diagnosis of rhabdomyosarcoma. While training on 274 patients and testing on an external test set of 30 patients, they found an AUC‐ROC of 0.64 for clear cell sarcoma, 0.89 for alveolar rhabdomyosarcoma, and 0.61 for embryonal rhabdomyosarcoma.

Zhang et al. [58] also focused on diagnosing subtypes of rhabdomyosarcoma. They found an accuracy of 84% for alveolar rhabdomyosarcoma, 90.2% for embryonal rhabdomyosarcoma, and 76.3% spindle cell sclerosing rhabdomyosarcoma. They also tried to predict prognosis based on the pathological images. They found, that when using this image‐based prognostic score to predict patient risk for relapse, the ratio of patients in the image‐based high‐risk group was 4.64 times higher than the low‐risk group.

While the previous rhabdomyosarcoma studies used 20× or 40× magnification, Agarwal et al. [59] tried to classify rhabdomyosarcoma subtypes with a magnification of only 5×. By doing so, they intend to decrease training time and to enhance processing, by decreasing the amount of data per patient. Their method, combining multiple trained DL methods in an ensemble, yielded a 95% classification accuracy.

Milewski et al. [60] did extensive research to predict several genetic mutations from hematoxylin and eosin (H&E) images for rhabdomyosarcoma patients, after which they trained a DL method to predict event‐free survival from whole slide images. They found that their method predicted event‐free survival better than current clinical models.

Rather falling into the area of translational cytology than histopathology, Berker et al. [61] used a process called transfer learning to introduce a method of image‐based phenotypic drug profiling with the aim of better classifying drug response in vivo. They aimed to predict whether wells contained viable cells or not. They pretrained a CCN via Imagenet and phases of fine‐tuning to assess tumor cell viability from confocal fluorescence microscopy images of spheroids in response to treatment modalities. They used available cell lines (e.g., one neuroblastoma cell line) and isolated patient cells with the aim to provide an image‐based drug response tool for individualized, more effective treatment of pediatric cancer patients.

#### 4.2.2 Segmentation

One study was focused on segmentation. van der Kamp et al. [62] annotated eight selected cell types on the HE‐stained slides of Wilms tumor patients, and trained a DL method to automate this segmentation process. They found an overall Dice score of 0.85, with the lowest Dice score for areas of bleeding and the highest Dice score for areas of necrosis (Tables 3 and 4).

**TABLE 3:** Table of inclusions for pathological studies.

Title	First author	Year	Task	Clinical focus	Staining	Magnification	Tumor type
Pathological Prognosis Classification of Patients With Neuroblastoma Using Computational Pathology Analysis	Liu	2022	Clas	Favorable/unfavorable	H&E	40×	Neuroblastoma
Convolutional Deep Belief Network With Feature Encoding for Classification of Neuroblastoma Histological Images	Gheisari	2018	Clas	Differentiated/undifferentiated and subtypes	H&E	N.S.	Neuroblastoma
DetexNet: Accurately Diagnosing Frequent and Challenging Pediatric Malignant Tumors	Liu	2020	Clas	Subtypes	Not specified	40×	Peripheral neuroblastic tumors
Machine Learning for Rhabdomyosarcoma Histopathology	Frankel	2022	Clas	Subtypes	H&E	40×	Soft tissue sarcomas
Deep Learning of Rhabdomyosarcoma Pathology Images for Classification and Survival Outcome Prediction	Zhang	2022	Clas	Subtypes	H&E	20×	Rhabdomyosarcoma
Rhabdomyosarcoma Histology Classification Using Ensemble of Deep Learning Networks	Agarwal	2020	Class	Subtypes	H&E	5×	Rhabdomyosarcoma
Predicting Molecular Subtype and Survival of Rhabdomyosarcoma Patients Using Deep Learning of H&E Images	Milewski	2023	Pred	Event‐free survival	H&E	40×	Rhabdomyosarcoma
Patient‐by‐Patient Deep Transfer Learning for Drug‐Response Profiling Using Confocal Fluorescence Microscopy of Pediatric Patient‐Derived Tumor‐Cell Spheroids	Berker	2022	Clas	Cell Death/no cell death	Hoechst 33342	N.S.	Brain/sarcoma/neuroblastoma
Automated Deep Learning‐Based Classification of Wilms Tumor Histopathology	van der Kamp	2023	Seg	Renal tissue and tumor subtypes	H&E	20×	Wilms tumor

Abbreviations: Clas, classification; H&E, hematoxylin and eosin; N.S., not specified; Pred, prediction; Seg, segmentation. The greyish color supports the visual differentation of the different tasks (Classification, Prediction, etc…).

**TABLE 4:** Table of inclusion for miscellaneous studies.

Title	First author	Year	Image type	Task	Clinical focus	Tumor type
Explainable AI for Retinoblastoma Diagnosis: Interpreting Deep Learning Models With LIME and SHAP	Aldughayfig	2023	Fundoscopy	Clas	Retinoblastoma/no retinoblastoma	Retinoblastoma
Automatic Retinoblastoma Screening and Surveillance Using Deep Learning	Zhang	2023	Fundoscopy	Clas	Normal fundus/stable retinoblastoma/active retinoblastoma	Retinoblastoma
EyeScreen: Development and Potential of a Novel Machine Learning Application to Detect Leukocoria	Bernard	2023	Smartphone pictures	Clas	Leukocoria/no leukocoria	Retinoblastoma
Improving Artificial Intelligence‐Based Diagnosis on Pediatric Skin Lesions	Mehta	2023	Skin pictures	Clas	Melanoma/other skin lesions	Melanoma
Semi‐Supervised Segmentation of Retinoblastoma Tumors in Fundus Images	Rahdar	2023	Fundoscopy	Seg	Tumor	Retinoblastoma

Abbreviations: Clas, classification; Seg, segmentation. The greyish color supports the visual differentation of the different tasks (Classification, Prediction, etc…).

### 4.3 Deep Learning in Image‐Based Diagnostics

Five studies about DL for applications in image‐based diagnostics were included. Most studies (*n* = 4) focused on classification, with one on segmentation.

#### 4.3.1 Classification

Two studies focused on the classification of fundoscopic images. First, Aldughayfiq et al. [63] used 400 fundoscopic images with retinoblastoma and 400 images without retinoblastoma to develop a classification method, with a final accuracy of 97%. A significant component of their work was explainable AI (XAI), for which they used two techniques to visualize which parts of the image were most decisive for the classification of the specific image [63]. In line with clinical experience, the DL methods seemed to use features like yellow‐white masses and calcifications to predict the classification outcome.

Zhang et al. [64] also focused on classification of fundoscopic images, but with the aim of surveillance in retinoblastoma instead of diagnostics. Using a large dataset for training and an external test set of 103 patients for testing they showed an accuracy of 99% for the classification between normal and active retinoblastoma and 93% for the classification between stable and active retinoblastoma. In addition to the technical evaluation, they performed a clinical analysis in a simulated scenario where the DL method and the ophthalmologist worked together to perform the diagnosis, in which performance of the ophthalmologist seemed to improve.

The last paper about retinoblastoma screening, by Bernard et al. [65], used smartphone pictures to classify leukocoria, a telltale sign of retinoblastoma. In a low‐income country, they set up a workflow to test their developed classification method, in the form of a smartphone app. In their test set of 291 patients, their method scored a sensitivity of 87% and a specificity of 73%.

Mehta et al. [66] used both an adult and pediatric dataset to train a DL method for classification of melanoma on pictures of pediatric skin lesions. The classification method developed on a dataset with additional pediatric patients showed an increased performance compared with the method trained on only adult patients.

#### 4.3.2 Segmentation

The study by Rahdar et al. [67] focused on segmentation of fundoscopic images. They used a large dataset containing more than 4000 fundoscopic images to perform non‐DL‐based, unsupervised segmentation. These segmentations were manually checked for quality and either excluded or edited, after which the obtained segmentations were used to train a DL method for retinoblastoma segmentation, scoring a final average Dice of 0.93.

## 5 Discussion

Our review focused on DL in medical images in pediatric surgical oncology. We chose to focus on DL in contrast to classical machine learning, because of the rapid growth of DL‐based image research, the high applicability of DL in image specific applications and its potential to create insights beyond human comprehension. In total, we included 36 studies in our review. Most of our included studies were published in 2023 (*n* = 14), with the oldest studies dating back to 2017, indicating the recent development of this area in the field of pediatric oncology.

Four types of DL‐specific tasks were identified in our included studies. First, classification tasks were most common, with studies classifying between malignant and benign or with studies doing multiclass classification for cancer specific subtypes. Prediction was only present in one article, focused on predicting dose distributions in radiotherapy. Other studies focused on segmentation, mainly for radiology and pathology. These studies mainly segmented tumor, but segmentation of healthy structures was also an area of research. Lastly, a task present only in radiology was image synthesis. For image synthesis all studies were embedded within radiation therapy, with studies trying to produce synthetic CT images for radiotherapy planning from either MRI or CBCT. The research in adult oncology covers a wider range of technical tasks than the studies we covered in our review. The biggest category of technical tasks missing is image registration, in which different images are aligned together [68]. For example, this can be used to register per‐operative US images to the pre‐operative MR imaging. Since registration is a relatively new field in DL, it is expected that this research will follow in the coming years.

We decided to divide our review into three chapters, reflecting three important sources of visual information in clinical practice. Most of the included studies were about radiology and nuclear imaging (*n* = 22). In adult oncology, this category is also of big importance [16]. Pathology was an important aspect of our review (*n* = 9). Again, this is a large area of research in adult oncology as well, with more than 40 studies included in a recent review by Ahmed, Abouzid, and Kaczmarek [69]. In our last category, DL in other medical images, we only identified studies about fundoscopy and skin pictures. An area especially important for pediatric surgeons, DL in endoscopic and laparoscopic images, is lacking in our review but abundant in adult oncology. For example, DL can be used to classify polyps during colonoscopy, or to perform landmark detection in laparoscopic surgery [17]. In the future years, when laparoscopic surgery in children with solid tumors might be more common, this might be an interesting field of research.

It is tempting to make statements about the general performance of the DL methods: But even for comparing performance in similar tasks, the spread of tumor types and (clinical) application is too big. Moreover, the performance metrics we talked about in the review are a mere proxy for clinical implementation. All research focused on DL should consider translation to the clinical workflow and choose the performance metrics accordingly [70]. Questions about the effect of false negatives and false positives should be asked to make a trade‐off between sensitivity and specificity, for example. A Dice score should not be used without knowing what the clinical effect of a slightly too big or a slightly too small segmentation would be. Is the segmentation always slightly too big or too small or is this inconsistent? Questions like these can only be asked and answered by those with clear understanding about both DL and the clinical practice.

Of course, it is essential to not only think about the validation of the algorithm but also consider the effect of the algorithms on the clinical workflow and the individual patient. For this, it is important to consider the use case of the DL application, does it aid or augment the clinical specialist? How does the clinical practice change when this application is implemented in the workflow? For example, some studies focused on screening in hospitals without specialized personal or low‐income countries [31, 65]. Other studies focused on aiding the clinical specialist, for example in tumor subtyping or volume measurements [45]. Lastly, some research was aimed at augmenting the specialist in tasks currently not part of the clinical workflow, for example in predicting based on imaging which patients would respond to chemotherapy [39]. Consequently, the place and task of the DL method in the clinical workflow determines the potential risks and benefits. Some of the included studies did look at the potential impact of their applications. For example, Florkow et al. created radiotherapy plans with‐ and without DL enabled skeleton growth center avoidance to show the potential benefits of their method [51]. To our knowledge, no DL methods specific for pediatric oncology are currently approved for clinical use. In adult oncology, several applications have already made the transition from research settings to clinical settings [20, 71]. Tools such as the APPRAISE‐AI tool can help assess the clinical safety and utility of AI algorithms [72].

The best path forward for DL in pediatric oncology depends on the application. It begins with identifying clear use cases with input from all relevant stakeholders, including pediatric oncologists, radiologists, pathologists, and radiotherapists. This is followed by collecting and labeling appropriate data. Due to the rarity of pediatric oncology cases, multicenter studies are essential, though regulatory, ethical, and political challenges can arise. Federated learning, a method of training DL applications without the need for sharing of data, offers a potential solution to these limitations [73, 74]. Clinical validation, ideally conducted across multiple centers and embedded in larger studies like UMBRELLA is critical for implementing DL models in practice [9]. Generalizability, how well an algorithm performs with diverse data, machines, or settings, is crucial, especially during validation and implementation. Rigorous, multicenter studies are needed to incorporate DL into clinical guidelines, with the validation method tailored to the use case. DL aimed to augment the specialist should be validated like all new clinical tests or applications: with randomized controlled trials being the gold standard.

Trustworthy DL is of uttermost importance throughout the whole process of implementing DL in the clinical practice. Logically, this starts with trustworthy research with transparent reporting about research population, data quality, and performance. Guidelines such as STREAM‐URO, created for reporting ML in urology, should be developed specifically for pediatric oncology The type of validation that was used in the articles varied widely. Some articles used cross validation, other articles used an internal or external test set. Some articles reported selection on data quality, which might have impacted the validation [64]. Due to the small number of patients and the big variety in between patients, validation is an extra challenge in the pediatric population. Assessing all articles for their validation methods, risk of bias and other important limitations falls outside our aim to provide a general overview of the field.

Generalizability of the DL method will be key for implementing the developed methods outside the developing center. As data quality is of high importance for this, data and knowledge sharing is key to create applicable DL solutions. Data standardization and multicenter studies are key for this. Another factor that can help in building trust in DL methods is explainable AI (XAI). XAI aims to increase the interpretation of the DL algorithm, which is thought to increase trust [75, 76].

While this study showed that the development of DL in pediatric oncology is still quite limited, the next steps forward should already be considered. Again, the adult oncology can guide our field, as plenty of studies about the validation of DL in the clinical workflow have been performed, together with clear guidelines and roadmaps to go forward [77, 78, 79, 80, 81, 82, 83]. However, pediatric research has unique challenges: the vulnerability of pediatric patients necessitates special ethical considerations, and the rarity of cases complicates data availability and validation. Pediatric oncology should adapt adult oncology frameworks where feasible, while proactively addressing pediatric‐specific challenges.

## 6 Conclusion

This review provided an overview of DL research on image‐based applications in pediatric surgical oncology. Current experimental algorithms in imaging, pathology, and other fields of clinical imaging show promising performance. However, more research is needed to interpret these results and their effect on clinical practice. The more advanced status of DL in adult oncology should be used to guide researchers in the pediatric field forward. While we are at the start of the DL revolution, we are not even close to revealing the full potential of DL applications: giving insights beyond human comprehension and leading to further improvement of the outcomes of children with cancer.

## Author Contributions

**M. A. D. Buser:** conceptualization (lead), data curation (lead), formal analysis (lead), investigation (lead), methodology (lead), project administration (lead), software (lead), validation (supporting), visualization (lead), writing – original draft (lead). **J. K. van der Rest:** conceptualization (equal), data curation (equal), methodology (equal), project administration (equal), software (equal), writing – review and editing (supporting). **M. H. W. A. Wijnen:** conceptualization (supporting), methodology (supporting), supervision (lead), writing – review and editing (equal). **R. R. de Krijger:** supervision (equal), writing – review and editing (equal). **A. F. W. van der Steeg:** conceptualization (supporting), methodology (supporting), supervision (lead), writing – review and editing (lead). **M. M. van den Heuvel‐Eibrink:** conceptualization (supporting), supervision (supporting), writing – review and editing (equal). **M. Reismann:** conceptualization (supporting), investigation (supporting), methodology (supporting), writing – review and editing (supporting). **S. Veldhoen:** supervision (equal), writing – review and editing (equal). **L. Pio:** conceptualization (supporting), writing – review and editing (equal). **M. Markel:** conceptualization (lead), formal analysis (lead), investigation (lead), methodology (lead), writing – original draft (supporting), writing – review and editing (lead).

## Ethics Statement

This study was conducted in accordance with the Declaration of Helsinki. Ethics approval was not required for this study as it did not involve human subjects.

## Conflicts of Interest

The authors declare no conflicts of interest.

## Supporting information


**Table S1.** Search strings.

## Data Availability

All data analyzed during this study are included in this article and its Supporting Information.
