# Supplementary material for: Deep Learning and Multidisciplinary Imaging in Pediatric Surgical Oncology: A Scoping Review
Source: Cancer Med. 2025 Jan 15;14(2):e70574. doi: 10.1002/cam4.70574 (PMC11733598; doi:10.1002/cam4.70574)
Supplement: Supplementary file 1 — Table S1. Search strings. [file CAM4-14-e70574-s001.docx]

Appendix A

Table S1: search strings

| **PubMed** | (("Artificial Intelligence"[Mesh] OR Artificial Intelligen*[TIAB] OR CNN[TIAB] OR Computer Vision[TIAB] OR Machine Learn*[TIAB] OR Deep Learn*[TIAB] OR Deep-Learn*[TIAB] OR neural network*[TIAB] OR AI[TIAB]))  AND (("Child"[Mesh] OR Child*[TIAB] OR "Pediatrics"[Mesh] OR Pediatric*[TIAB] OR Paediatric*[TIAB] OR infant*[TIAB]))  AND (("Neoplasms"[Mesh] OR Neoplasm*[TIAB] OR Cancer*[TIAB] OR oncolog*[TIAB] OR tumor*[TIAB] OR tumour*[TIAB] OR Malignan*[TIAB])) |
| --- | --- |
| **Scopus** | TITLE-ABS-KEY ( "Artificial Intelligen*" OR "CNN*" OR "Computer Vision" OR "Machine Learn*" OR "Deep Learn*" OR "Deep-Learn*" OR "neural network*" OR "AI")  AND TITLE-ABS-KEY ( "Child*" OR "Pediatric*" OR "Paediatric*" OR "infant*" )  AND TITLE-ABS-KEY ( "Neoplasm*" OR "Cancer*" OR "oncolog*" OR "tumor*" OR "tumour*" OR "Malignan*" ) |
| **Embase** | ('artificial intelligence'/exp OR 'artificial intelligen*':ab,ti,kw OR 'CNN':ab,ti,kw OR 'computer vision':ab,ti,kw OR 'machine learn*':ab,ti,kw OR 'deep learn*':ab,ti,kw OR 'Deep-learn*':ab,ti,kw OR 'neural network*':ab,ti,kw OR 'AI':ab,ti,kw)  AND ('child'/exp OR 'child*':ab,ti,kw OR 'pediatric'/exp OR 'pediatric*':ab,ti,kw OR 'paediatric*':ab,ti,kw OR 'infant*':ab,ti,kw)  AND ('malignant neoplasm'/exp OR 'neoplasm*':ab,ti,kw OR 'cancer':ab,ti,kw OR 'oncolog*':ab,ti,kw OR 'tumor*':ab,ti,kw OR 'tumour*':ab,ti,kw OR 'malignan*':ab,ti,kw) |
